# Vitamin C and E supplementation does not affect heat shock proteins or endogenous antioxidants in trained skeletal muscles during 12 weeks of strength training

**DOI:** 10.1186/s40795-017-0185-8

**Published:** 2017-08-17

**Authors:** K. T. Cumming, T. Raastad, A. Sørstrøm, M. P. Paronetto, N. Mercatelli, I. Ugelstad, D. Caporossi, G. Paulsen

**Affiliations:** 1 Department of Physical Performance, Norwegian School of Sport Sciences, Oslo, Norway; 2 Department of Movement, Human and Health Sciences, University of Rome “Foro Italico”, Rome, Italy; 3 Norwegian Olympic Sports Center, Oslo, Norway

**Keywords:** Stress proteins, Gene expression, Resistance training

## Abstract

**Background:**

Supplementation with large doses of antioxidants, such as vitamin C and E, has been shown to blunt some adaptations to endurance training. The effects of antioxidant supplementation on adaptations to strength training is sparsely studied. Herein we investigated the effects of vitamin C and E supplementation on acute stress responses to exercise and adaptation to traditional heavy load strength training.

**Methods:**

In a double blind placebo-controlled design, twenty-eight, young, trained males and females were randomly assigned to receive either vitamin C and E (C: 1000 mg, E: 235 mg, per day) or placebo supplements, and underwent strength training for 10 weeks. After five weeks, a subgroup conducted a strength training session to investigate acute stress responses. Muscle samples were obtained to investigate changes in stress responses and in proteins and mRNA related to the heat shock proteins (HSPs) or antioxidant enzymes.

**Results:**

The acute responses to the exercise session revealed activation of the NFκB pathway indicated by degradation of IκBα in both groups. Vitamin C and E supplementation had, however, no effects on the acute stress responses. Furthermore, ten weeks of strength training did not change muscle αB-crystallin, HSP27, HSP70, GPx1 or mnSOD levels, with no influence of supplementation.

**Conclusions:**

Our results showed that although vitamin C and E supplementation has been shown to interfere with training adaptations, it did not affect acute stress responses or long-term training adaptations in the HSPs or antioxidant enzymes in this study.

**Electronic supplementary material:**

The online version of this article (doi:10.1186/s40795-017-0185-8) contains supplementary material, which is available to authorized users.

## Background

Increased muscle strength and mass by strength training are important for both physical performance and good health [1]. Adaptations to strength training includes both structural and biochemical changes in the muscle. The biochemical adaptations include changes in systems that deal with stress and support restitution after exercise, such as the heat shock proteins (HSPs) and the endogenous antioxidants. HSPs have the ability to protect against various cellular stressors by preventing protein damage and restoring the function of already damaged/unfolded proteins [2]. As a response to high intensity strength exercise, HSPs bind to and accumulate in damaged structures [3, 4]. This accumulation of HSPs correlates with the decline in force generating capacity [4, 5], which suggests that increased HSPs levels in muscle could potentially improve the recovery process and restore muscle function. Increased basal levels of HSPs have been observed after 5–11 weeks of strength training in both lower and upper body muscles [6, 7]. Increased intracellular HSP by training could potentially increase the rate of regeneration and training adaptations.

In addition to the HSPs, eukaryotic cells have developed its own antioxidant enzyme system to support optimal redox regulation. Such systems include glutathione peroxidases (GPx) and superoxide dismutases (SOD), which both have the ability to reduce H_2_O_2_ to a less harmful molecule. These enzymes has previously been shown to increase by both endurance and strength training [8–10]. Many athletes use antioxidant supplements [11] in the belief they could further protect against exercise induced stress and thus improve recovery. However, recent studies indicate that antioxidant supplementation have the ability to blunt training adaptations in skeletal muscle [12, 13]. A proposed mechanism for the observed interference between antioxidant supplementation and training adaptations has been related to changes in redox status in the muscle, which in turn might affect cell signaling [14]. One potential target for antioxidant supplementation is the stress- and redox-sensitive NFκB pathway, which upon stimulation activates transcription of a range of stress related proteins, such as HSPs and endogenous antioxidants [15–17]. Consequently, supplementation with large doses of antioxidants could potentially reduce the physiological up-regulation of cellular defensive systems (HSPs and endogenous antioxidant enzymes) upon regular exercise stress. Vitamin C supplementation alone has been shown to increase basal protein levels of HSP70 in skeletal muscle, and blunt HSP70 protein expression in human lymphocytes exposed to oxidative stress [18]. Furthermore, ingestion of vitamin C and E decreases HSP70 mRNA levels after endurance exercise [19]. On the other hand, antioxidant supplementation has been shown to blunt the upregulation of *SOD2* and *GPx1* mRNAs induced after a period of endurance and strength training [20]. Undesirable effects of antioxidant supplementation on protein levels of GPx1 or mnSOD after endurance training was, however, not observed in two later studies [21–23]. Nevertheless, the apparent interference between antioxidant supplements and HSPs and endogenous antioxidants, could potentially affect the training induced adaptations in these important proteins.

We have previously shown that vitamin C and E supplementation did not have an effect on HSP levels in response to 11 weeks of endurance training [24]. To our knowledge, adaptation mechanisms in the HSPs, and GPx1 and mnSOD, in response to heavy load strength training in combination with vitamin C and E supplementation has not been investigated yet in humans. Thus, the aim of the present study was to investigate the effect of vitamin C and E supplementation on both acute exercise induced mRNA expression and long-term training adaptations in protein levels of HSPs and endogenous antioxidants during a period of heavy-load strength training.

Despite an increase in muscle mass over 10 weeks of training, we have reported that vitamin C and E supplementation blunted hypertrophy related signaling to the strength training session investigated in the present study [25]. Based on these results, we hypothesized that vitamin C and E supplementation would be able to blunt the stress induced by exercise, measured as reduced activation of NFκB pathway, and expression of HSPs (*CRYAB* and *HSPB1*) or antioxidant enzymes (*SOD2* and *GPx1*). Further, we hypothesized that vitamin C and E supplementation would blunt the long term (10 weeks) training induced increases in the heat shock proteins (αB-crystallin, HSP27 and HSP70) and endogenous antioxidant enzymes (GPx1 and mnSOD).

## Methods

### Participants

Eighteen male and ten female participants (*n* = 28; age 25 ± 5 years, height 175 ± 8 cm, body weight 74 ± 13 kg) completed the study. All participants were physical active, accustomed to strength training, conducting regularly strength training 1–4 times per week before the start of the study. Physical activity prior to the study was reported with a questionnaire. All participants gave written informed consent before entering the study, and were informed about potential risks related to the experiment. The study was approved by the Regional Ethics Committee of Southern Norway (2010/1352) and was performed in accordance with the Helsinki Declaration.

### Experimental design

The detailed experimental design and limitations of the present study has already been described [26]. Results on muscle mass and more strength measurements have been published previously [25]. In a randomized double blinded manner, participants were allocated to receive either a vitamin C and E or a placebo supplement based on baseline maximal strength tests (1RM) and sex.

### Supplements

The vitamin C and E and placebo pills were produced by Petefa AB (Västra Frölunda, Sweden) under Good Manufacturing Practice (GMP) requirements. Each vitamin pill contained 250 mg of ascorbic acid and 58.5 mg DL-alpha-tocopherol acetate. The placebo pills had the same shape and appearance as the vitamins pills. All supplements were stored in similar unlabeled boxes, and were consumed orally with an artificially favored sucrose (30 g) drink to mask any potential taste from the pills.

The participants ingested 2 pills (total 500 mg of vitamin C and 117 mg vitamin E) 1–3 h before every training session and 2 pills in the first hour after training. On non-training days, the participants ingested 2 pills in the morning and 2 pills in the evening. The intake of pills was confirmed with an online training diary. Thus, daily dosage was 1000 mg of vitamin C and 235 mg vitamin E. The total supplemental dosage of vitamin C was ~13 times higher and ~23 times higher for vitamin E than the recommended daily dietary allowance in the Nordic countries.

Beside the supplementation given in the study, the participants were asked to not take any form of nutritional supplement or medication that could affect the strength training adaptations, such as NSAIDs. They were also asked not to drink more than 2 glasses of juice and 4 cups of coffee or tea per day. Juices rich in antioxidants, such as grape juice, were completely avoided.

### Training

The exercise consisted of strength training with heavy loads (6-11RM) for 10 weeks. The first six weeks the loads were 3 × 9-11RM, and 3-4 × 6-8RM the last four weeks. Sets were separated by a 1–1.5 min break. The exercise program included exercises for all major muscle groups in a 4-split exercise program (two upper- and two lower body sessions per week), with a seven upper and six lower body exercises. The exercise program was designed with the main goal to stimulate both maximal strength and muscle growth. The adherence and control of exercise and supplementation was monitored and logged using an online training diary. For variation and motivation participants were allowed to do alternative exercise forms (e.g. cycling or cross country skiing) once per week in addition to the planned training sessions.

### Acute exercise session

After 4–6 weeks, ten male and five female participants (*n* = 15; age 26 ± 7 years, height 177 ± 7 cm, body weight 73 ± 13 kg) volunteered to an acute exercise experiment. The exercise session consisted of 4x10RM of leg press and knee-extension, with 1 min rest between sets and 3 min rest between exercises. Muscle biopsies were collected from *m. vastus lateralis* before and 100 and 150 min after the standardized exercise session using a modified Bergström technique (described in the *Muscle tissue sampling and pre-analytic handling*). Participants ingested the supplements, vitamin C and E or placebo, together with a standardized breakfast (3 g oat per kg body weight boiled in water with 5 g sucrose) two hours before the exercise session. A second dose of supplements was taken immediately after the exercise session.

### Muscle tissue sampling and pre-analytic handling

Muscle biopsies from the mid-portion of the right *m. vastus lateralis* were collected before and after the training intervention. The post training insertion was proximally located to the pre-training site (approximately 3 cm). For the participants that also took part in the mid-way, acute session experiments, the biopsies were collected from the left *m. vastus lateralis*. One insertion was made for the pre sample, and a new insertion was made proximally from this for the post exercise samples. The two post samples were collected from the same insertion site, at two different directions. The first was sampled proximally and the second distally from the insertion site. The procedure was conducted under local anesthesia (Xylocain adrenalin, 10 mg/ml + 5 μg/ml, AstraZeneca PLC, London, UK). Approximately 200 mg (2-3 × 50–150 mg) of muscle tissue was obtained with a modified Bergström-technique. Tissue intended for homogenization and protein measurements was quickly washed in physiological saline, and fat, connective tissue, and blood were removed and discarded before the sample was weighed and quickly frozen in isopentane cooled on dry ice. Tissue intended for mRNA analyses were placed in RNAlater (AM7020, Ambion, Life technologies, Carlsbad, CA, USA). All muscle samples were stored at −80 °C for later analyses.

### Protein immunoblot

Muscle tissue was homogenized using a commercial homogenization buffer (78510, T-PER/Tissue Protein Extraction Reagent, Thermo Scientific, Rockford, IL, USA) with a cocktail of protease and phosphatase inhibitors (1861281, Halt protein and phosphatase inhibitor cocktail, Thermo Scientific) and EDTA (1861274, Thermo Scientific). Quantification of protein extracts was assessed with the BioRad DC protein micro plate assay (0113, 0114, 0115, Bio-Rad, CA, USA). A filter photometer (Expert 96, ASYS Hitech, Cambridge, UK) was used to measure the colorimetric reaction and sample protein concentration was calculated by the provided software (Kim, ver. 5.45.0.1, Daniel Kittrich, Prague, Czech Republic).

Extracted proteins were analyzed by western blotting. Equal amounts of protein were loaded per well (15 μg) and separated by 4–12% SDS gradient gels under denaturized conditions. Proteins were transferred onto PVDF membranes (162–0177, Immuno-blot, Bio-Rad or iBlot Gel transfer stacks, IB4010, Invitrogen, Carlsbad, CA, USA) before blocked in a 5% fat free skimmed milk and 0.1% TBS-t solution (TBS, 170–6435, Bio-Rad; Tween 20, 437082Q, VWR International, Radnor, PA, USA; Skim milk, 1.15363, Merck, Darmstadt, Germany). Blocked membranes were incubated in antibodies against GPx1 (ab22604, Abcam, Cambridge, UK), mnSOD (ab16956, Abcam), IκBα (ab32518, Abcam), HSP70 (ADI-SPA-810, Enzo Life Sciences, Farmingdale, NY, USA) or αB-crystallin (ADI-SPA-222, Enzo Life Sciences), followed by incubation in an appropriate secondary antibody (31430, Thermo Scientific; 7074, Cell Signaling Technology, Danvers, MA, USA). Between stages, membranes were washed in 0.1% TBS-t solution. Bands were visualized using a HRP-detection system (34076, Super Signal West Dura Extended Duration Substrate, Thermo Scientific). Chemiluminescence was measured using a CCD image sensor (Image Station 2000R or Image Station 4000R, Eastman Kodak Inc., Rochester, NY, USA) and band intensities were calculated with the Carestream molecular imaging software (Carestream Health Inc., Rochester, NY, USA). All samples were run as duplicates and mean values were used for statistical analyses.

### ELISA

HSP27 in the cytosolic and cytoskeletal fractions was measured as previously described in detail [5]. Briefly, HSP27 was detected using a in house-made double antibody sandwich ELISA. By using capture antibodies (25 ng/well; ADI-SPA-800, Enzo Life Sciences) and detection antibodies against HSP27 (ADI-SPA-803, Enzo Life Sciences), HSP27 was determined by using a filter photometer (Expert 96, ASYS Hitech) measuring optical density at 450 nm.

### RT-qPCR

Total RNA was extracted from muscle biopsies (*n* = 14) from the acute study by homogenization in TRIzol reagent (15596, Invitrogen, Life Technologies). DNase I digestion was performed using RNase free-DNase from Qiagen (79254, Qiagen Inc., Germantown, MD, USA) in order to prevent genomic DNA contamination. Quantitative Reverse Transcription PCR (RT-qPCR) analysis was performed in an Applied Biosystems 7500 Real-Time PCR System (Applied Biosystems, Foster City, CA, USA) by using Power SYBR Green RNA-to-Ct™ 1-step Kit (4389986, Applied Biosystems) supplemented with forward and reverse primer in a total volume of 20 μl. The Reverse Transcription step was performed at 48 °C for 30 min in the presence of RNase inhibitor by using 6 ng of total RNA Thermocycling conditions were according to the recommendations of the manufacturer. *Ct* values for gene expression were calculated according to the comparative *Ct* method [27]. Relative quantification was performed by simultaneous quantification of *GAPDH* and *18S* gene expression. The primers used for RT–qPCR analyses can be found as Additional file 1.

### Statistics

All values are presented as means ± standard deviations (SD). A two-way ANOVA was used to evaluate the effects of training (time) and supplementation (interaction), and a Holm-Sidak multiple comparisons test was chosen for post hoc analyses. In general, figures display individual data points, mean and standard deviations. The level of significance was set to *P* < 0.05. Graphpad Prism 6 (GraphPad Software Inc., La Jolla, CA, USA) was used for the statistical analyses.

## Results

### Acute response to exercise

**Fig. 1:**
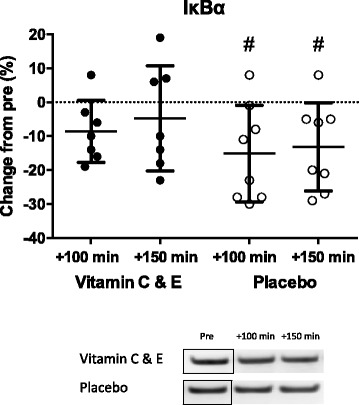
Relative changes in IκBα for the vitamin C and E- (filled circles; *n* = 7) and placebo group (open circles; *n* = 8) acutely after (+100 and 150 min) a standarized high intensity strength training session. Strippled line indicates baseline values. Panel shows representative protein immunoblots. Note that the bands from the pre-sample are rearranged to fit the panel. #: different from pre (*P* < 0.05)

**Fig. 2:**
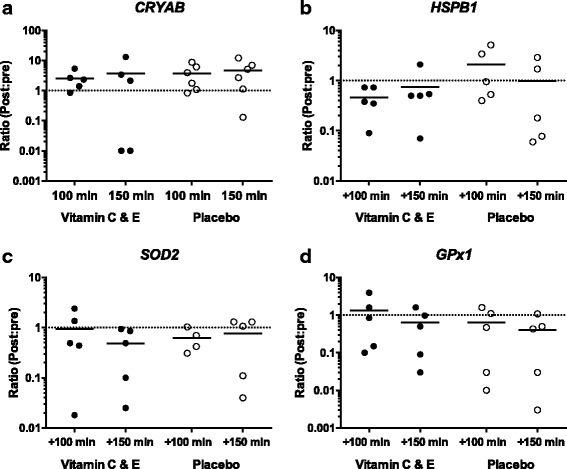
Changes (ratio between post and pre values) in mRNA expression of CRYAB (**a**), HSPB1 (**b**), SOD2 (**c**) and GPx1 (**d**) for the vitamin C and E- (filled circles; *n* = 5) and placebo group (open circles; *n* = 4–6) +100 and +150 mins after the standardized acute exercise session performed midway in the training intervention. Strippled line indicates baseline values

The p65 NFκB translocation suppressor IκBα was decreased in the placebo group at 100 and 150 min after the standardized strength training session (*P* < 0.05; Fig. 1), whereas no statistically significant changes were observed in the vitamin C + E group. However, no differences were observed between groups at any time points post exercise. The gene expression (mRNA content) of the small HSPs αB-crystallin (CRYAB) and HSP27 (HSPB1) was not different post exercise at any time point compared to before the exercise session for any of the groups (Fig. 2a-b). This was also true for the expression of mnSOD (SOD2) and GPx1 mRNA, with no statistically significant changes post exercise or between groups (Fig. 2c-d).

### Adaptation to training

**Fig. 3:**
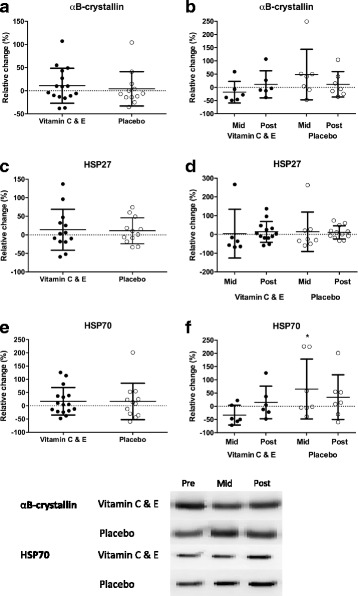
Relative changes in protein levels for αB-crystallin pre-post (**a**), αB-crystallin mid-post (**b**), HSP27 pre-post (**c**) HSP27 mid-post (**d**), HSP70 pre-post (**e**) and HSP70 mid-post (**f**) for the vitamin C and E- (filled circles; *n* = 6–16) and placebo group (open sircles; *n* = 7–12) after 10 weeks of strength training. Strippled line indicates baseline values. Panel shows representative protein immunoblots. Panel shows representative protein immunoblots. Note that the pre-mid-post figures only includes data from the participants that volunteered for the standardized acute exercise session performed midway in the training intervention. *: different compared to vitamin C and E group (*P* < 0.05)

**Fig. 4:**
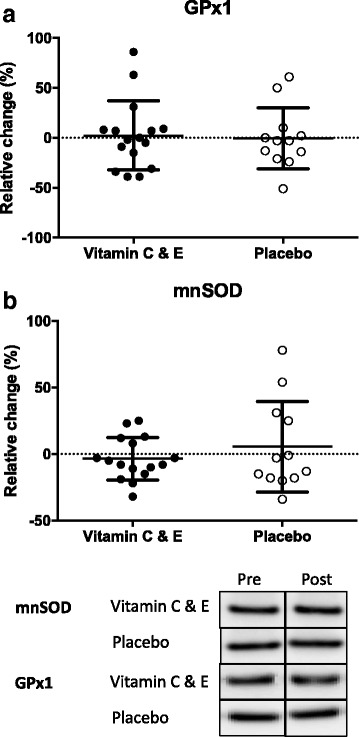
Relative changes in protein levels for GPx1 (**a**) and mnSOD (**b**) for the vitamin C and E- (filled circles; *n* = 16) and placebo group (open circles; *n* = 12) after 10 weeks of strength training. Strippled line indicates baseline values. Panel shows representative protein immunoblots. Panel shows representative protein immunoblots. Note that the bands are rearranged to fit this panel

After 10 weeks of strength training, no changes were observed in protein content of the HSPs αB-crystallin, HSP27 or HSP70 for any of the groups (Fig. 3). The same were observed for GPx1 and mnSOD, with no changes for any of the groups (Fig. 4). For the subpopulation who volunteered for the acute study, we were able to add a mid-training time-point. For these participants, no changes in any of the HSPs was observed between pre- to mid training or mid- to post training intervention (Fig. 3 b, d, f). At the mid-training time-point, the placebo group had significant higher HSP70 content (*P* = 0,035) compared to the group receiving vitamin C and E supplementation. However, these results must be interpreted with caution due to few participants (*n* = 7) included in this analysis.

## Discussion

By means of a double blind randomized placebo-controlled trial, we investigated the effects of vitamin C and E supplementation on the acute stress response to exercise and long-term strength training adaptations in HSPs and endogenous antioxidant enzymes in recreationally strength trained males and females. The acute stress response was indicated by the significant reductions of IκBα levels, suggesting increased activity in the NFκB pathway [28]. Contrary to our initial hypothesis, vitamin C and E supplementation did not blunt the IκBα response or the concomitant changes in HSP (*CRYAB* and *HSPB1*) and endogenous antioxidant (*SOD2* and *GPx1*) mRNA levels in response to a session of heavy-load strength exercise. Despite an increase in muscle strength and mass in upper- and lower body exercises in both groups [25], no changes were observed in protein levels in the investigated HSPs (αB-crystallin, HSP27 or HSP70) or endogenous antioxidants (GPx1 and mnSOD) after 10 weeks of heavy load strength training.

### Heat shock proteins

The acute exercise bout conducted midway into the training period did not induce statistically significant upregulation of the investigated HSP genes after the standardized exercise bout. This occurred even though the NFκB pathway was activated as indicated by the significant degradation of IκBα in both groups after exercise. In unstressed muscle cells IκBα is bound to NFκB. Upon stress, IκBα is degraded and released, thus promoting nuclear export of NFκB [28]. Activation of this pathway, per se and in combination with HSF1 and AP-1 activation, has been associated with increased HSP protein expression after isometric contractions [29]. Consequently, activation of NFκB pathway demonstrate that the standardized strength exercise stimulated sufficient strain on the investigated muscles, which normally would increase HSP mRNA. The lack of significant changes in HSP mRNA levels must, however, be interpreted with caution because the statistical power in these specific analyses was poor (low number of biopsies available for mRNA analysis).

There is always the risk that we missed any effects of the supplements on the acute exercise response by investigating a quite narrow time point after the standardized exercise session. Therefore, changes in basal levels of HSPs from before to after the training period might better reflect the chronic effects of antioxidant supplementation. However, our participants did not change the basal levels of HSPs at all over the 10 week long training period. This is in contrast to former strength training studies where increased HSP protein content is observed in previously untrained participants [6, 7]. The explanation could be that our participants were moderately trained prior to participating in the study, and, most likely, already had achieved high protein levels of HSPs. Indeed, the initial training status has an influential role for further increases in several HSPs [6, 30]. Thus, they had less potential to further increase their muscle HSP content. Nevertheless, vitamin C and E supplementation had no effect on basal levels of the investigated HSPs over the 10-week training and supplementation period in our participants. However, it is important to point out that we did not observe any training effects on HSP levels. This will make it difficult to observe any potential effects of the supplements. We cannot rule out that antioxidant supplementation would have any detrimental effects on untrained participants where increases in HSP levels would most likely occur in response to heavy strength training.

### Endogenous antioxidants

So far, limited number of studies has investigated adaptations in antioxidant enzymes in human skeletal muscles after strength training. The acute exercise bout conducted in the middle of the training period resulted in degradation of IκBα, which indicates NFκB-activation. As mentioned, this is one of the signaling pathways inducing increased endogenous antioxidant expression [15, 17]. Thus, we would expect an increased mRNA levels of *GPx1*, *SOD1* or *SOD2* after the exercise session. This was, however, not the case, but as previously mentioned the analyses of acute changes in mRNA expression levels suffered from poor statistical power. Nevertheless, the lack of altered acute expression levels from exercise was consistent with the observations of unaltered basal protein levels of GPx1 and mnSOD over 10 weeks of training. As for the HSPs, vitamin C and E supplementation did not affect mRNA levels of *GPx1*, *SOD1* (CuZnSOD) nor *SOD2* (mnSOD) after the acute exercise session, or the basal protein level of GPx1 and mnSOD over 10 weeks of training. In contrast to our results, Ristow et al. [20] reported blunting effects from vitamin C and E supplementation on mRNA levels of *GPx1*, *SOD1* and *SOD2* after 4 weeks of circuit training (combination of strength- and endurance training). However, these results seem trivial, since expression of these genes would most likely increase acutely after a high intensity exercise session rather than in the basal rested state. One likely explanation to the unaltered protein levels of the investigated antioxidant enzymes in the present study would be that the participants had well developed antioxidant systems. However, markers of oxidative damage increase also in well trained individuals after a single bout of strength training [31], meaning that these enzymes are capable to increase even in the well trained. However, large and systematic alterations in redox status is needed, most likely by more frequent and higher intensity training [32].

### Limitations

A limitation in the present study was the limited number of participants recruited to the acute experiment (*n* = 15). Thus, we must interpret results from this part of the study with some caution. Especially the mRNA analyses which has poor statistical power due to very few samples, ranging from four to six samples/participants. However, we choose to include these results as they might help shed some light to the effects on vitamin C and E supplementations on the presented mRNA and protein expression.

We choose to investigate the degradation of IκBα as a marker for activation of the NFκB pathway. This pathway is one of several that can activate or increase the expression of HSP or the endogenous antioxidants.

## Conclusions

Vitamin C and E supplementation did not affect the acute gene expression of the investigated HSPs and endogenous antioxidant enzymes after high-load resistance exercise. Nor did antioxidant supplementation affect basal protein levels of HSPs or endogenous antioxidants over 10 weeks of strength training in trained individuals. Although supplementation with high doses of vitamin C and E did not interfere with adaptations in the stress related proteins investigated in this study, the training effects on these proteins was also lacking. Therefore, it is difficult to delineate the precise effects of the supplements. However, others and we have shown interference with other training adaptations in skeletal muscle. Consequently, athletes should critically evaluate possible pros and cons when considering the use of antioxidant supplements.

## Additional file

Additional file 1: Table S1. Human primer sequences used for the RT-qPCR analyses. The genes listed encodes to following proteins: *CRYAB* = αB-crystallin; *HSPB1* = HSP27 protein 1; *SOD2* = superoxide dismutase 2 or mnSOD; *GPx1* = glutathione peroxidase 1. (DOCX 55 kb)
