# Supplementary material for: Vitamin C and E supplementation does not affect heat shock proteins or endogenous antioxidants in trained skeletal muscles during 12 weeks of strength training
Source: BMC Nutr. 2017 Aug 17;3:70. doi: 10.1186/s40795-017-0185-8 (PMC7050865; doi:10.1186/s40795-017-0185-8)
Supplement: Supplementary file 1 — Human primer sequences used for the RT-qPCR analyses. The genes listed encodes to following proteins: CRYAB = αB-crystallin; HSPB1 = HSP27 protein 1; SOD2 = superoxide dismutase 2 or mnSOD; GPx1 = glutathione peroxidase 1. (DOCX 55 kb) [file 40795_2017_185_MOESM1_ESM.docx]

| **Gene name (accession no.)** | **Sense** | **Antisense** |
| --- | --- | --- |
| *CRYAB* (NM_001885) | GTCAACCTGGATGTGAAGCA | TTTTCCATGCACCTCAATCA |
| *HSPB1* (NM_001540) | GGACGAGCTGACGGTCAAG | AGCGTGTATTTCCGCGTGA |
| *SOD2* (NM_000636) | CCCTGGAACCTCACATCAAC | GGTGACGTTCAGGTTGTTCA |
| *GPx1* (NM_000581) | ACGATGTTGCCTGGAACTTT | TCGATGTCAATGGTCTGGAA |

Additional file 1: Table 1 Human primer sequences used for RT-qPCR

The genes listed encodes to following proteins: *CRYAB* = αB-crystallin; *HSPB1* = HSP27 protein 1; *SOD2* = superoxide dismutase 2 or mnSOD; *GPx1* = glutathione peroxidase 1.
